# Comparison of Multiparametric MRI Scoring Systems and the Impact on Cancer Detection in Patients Undergoing MR US Fusion Guided Prostate Biopsies

**DOI:** 10.1371/journal.pone.0143404

**Published:** 2015-11-25

**Authors:** Ardeshir R. Rastinehad, Nikhil Waingankar, Baris Turkbey, Oksana Yaskiv, Anna M. Sonstegard, Mathew Fakhoury, Carl A. Olsson, David N. Siegel, Peter L. Choyke, Eran Ben-Levi, Robert Villani

**Affiliations:** 1 Icahn School of Medicine at Mount Sinai, New York, New York, United States of America; 2 Fox Chase Cancer Center, Philadelphia, Pennsylvania, United States of America; 3 Molecular Imaging Program, National Institutes of Health, Bethesda, Maryland, United States of America; 4 Hofstra North Shore LIJ School of Medicine, New Hyde Park, New York, United States of America; National Cancer Centre Singapore, SINGAPORE

## Abstract

**Introduction:**

Multiple scoring systems have been proposed for prostate MRI reporting. We sought to review the clinical impact of the new Prostate Imaging Reporting and Data System v2 (PI-RADS) and compare those results to our proposed Simplified Qualitative System (SQS) score with respect to detection of prostate cancers and clinically significant prostate cancers.

**Methods:**

All patients who underwent multiparametric prostate MRI (mpMRI) had their images interpreted using PI-RADS v1 and SQS score. PI-RADS v2 was calculated from prospectively collected data points. Patients with positive mpMRIs were then referred by their urologists for enrollment in an IRB-approved prospective phase III trial of mpMRI-Ultrasound (MR/TRUS) fusion biopsy of suspicious lesions. Standard 12-core biopsy was performed at the same setting. Clinical data were collected prospectively.

**Results:**

1060 patients were imaged using mpMRI at our institution during the study period. 341 participants were then referred to the trial. 312 participants underwent MR/TRUS fusion biopsy of 452 lesions and were included in the analysis. 202 participants had biopsy-proven cancer (64.7%) and 206 (45.6%) lesions were positive for cancer. Distribution of cancer detected at each score produced a Gaussian distribution for SQS while PI-RADS demonstrates a negatively skewed curve with 82.1% of cases being scored as a 4 or 5. Patient-level data demonstrated AUC of 0.702 (95% CI 0.65 to 0.73) for PI-RADS and 0.762 (95% CI 0.72 to 0.81) for SQS (p< 0.0001) with respect to the detection of prostate cancer. The analysis for clinically significant prostate cancer at a per lesion level resulted in an AUC of 0.725 (95% CI 0.69 to 0.76) and 0.829 (95% CI 0.79 to 0.87) for the PI-RADS and SQS score, respectively (p< 0.0001).

**Conclusions:**

mpMRI is a useful tool in the workup of patients at risk for prostate cancer, and serves as a platform to guide further evaluation with MR/TRUS fusion biopsy. SQS score provided a more normal distribution of scores and yielded a higher AUC than PI-RADS v2. However until our findings are validated, we recommend reporting of detailed sequence-specific findings. This will allow for prospectively collected data to be utilized in determining the impact of ongoing changes to these scoring systems as our understanding of mpMRI interpretation evolves.

## Background

Multiparametric MRI (mpMRI) provides high quality imaging of the prostate that results in improved cancer detection with a positive predictive value of 93% [1] and negative predictive value of 95% [2]. mpMRI has been shown to improve risk stratification, enhance patient counseling regarding treatment options, and ultimately assist with selection of ideal candidates for active surveillance and other therapies [3–6].

**Fig 1:**
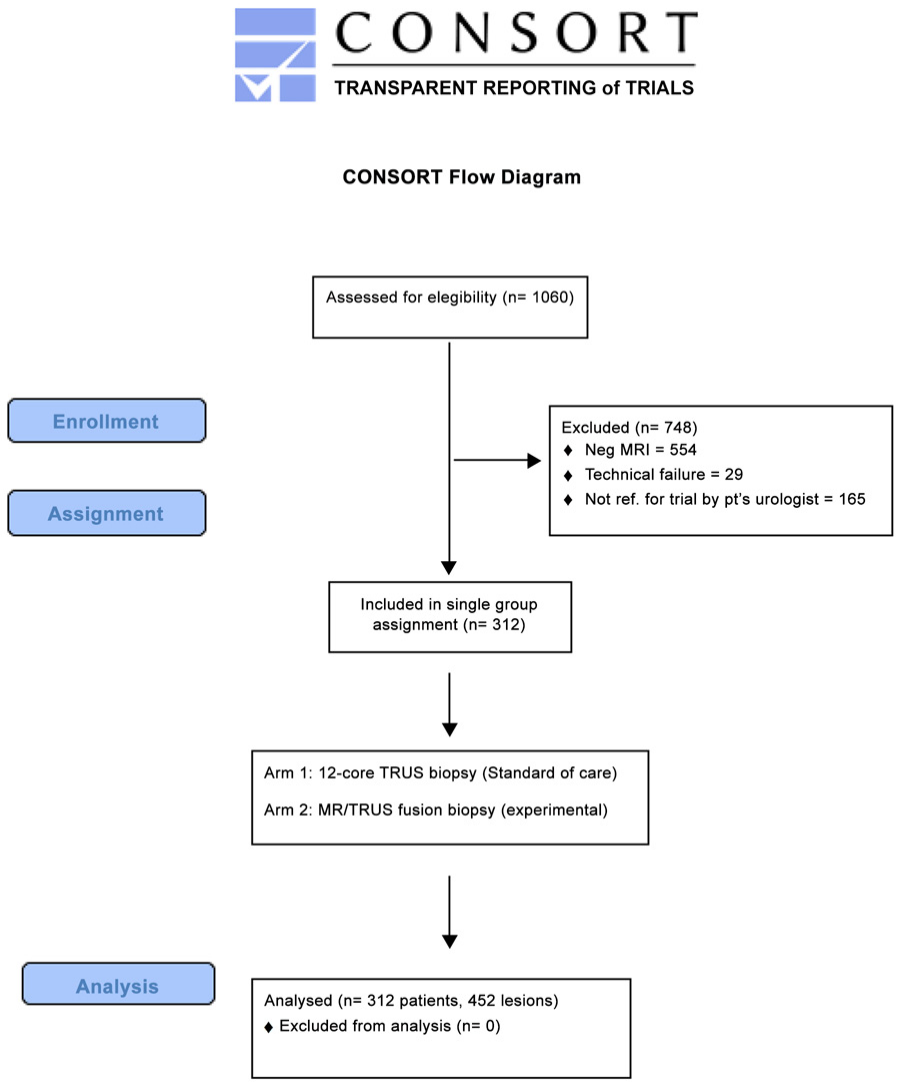
Consort Flow Diagram of trial from enrollment through analysis.

**Fig 2:**
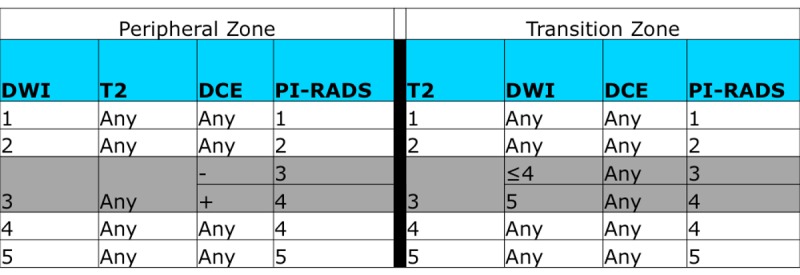
PI-RADS Scoring System.

Central to the utility of mpMRI as a diagnostic modality is an accurate, systematic, and reproducible scoring system for suspicious lesions. In 2012, the European Society of Urogenital Radiology (ESUR) published the Prostate Imaging Reporting and Data System (PI-RADS), a set of guidelines for structured MRI reporting which entails the application of a 5-point scoring system for each region with corresponding score for each sequence (T2, Diffusion-weighted imaging (DWI), Dynamic contrast enhanced (DCE), and MR-Spectroscopy). Due to the rapid adoption and utilization of mpMRI for the evaluation of prostate cancer, several limitations were identified with this scoring system, and a refined version of PI-RADS was developed in conjunction with the American College of Radiology. ACR PI-RADS v2 sought to establish technical parameters for prostate MRI; standardize terminology of reports; facilitate the use of MRI for targeted biopsy; aid in risk stratification; and enhance communication, quality assurance, and research[7, 8]. Overall ACR PI-RADS v2 assessment (henceforth simply referred to as “PI-RADS”) uses a 5-point scale based on the likelihood that a combination of MRI findings on DWI, T2W, and DCE correlates with the presence of a clinically significant cancer at a particular location in the prostate gland **(Figs 1 and 2)**.

**Fig 3:**
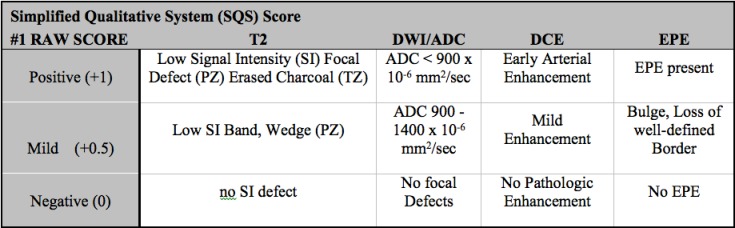
SQS Scoring System, Step 1: Calculate raw score by adding points for each finding listed. Score range is between 0–4.

**Fig 4:**
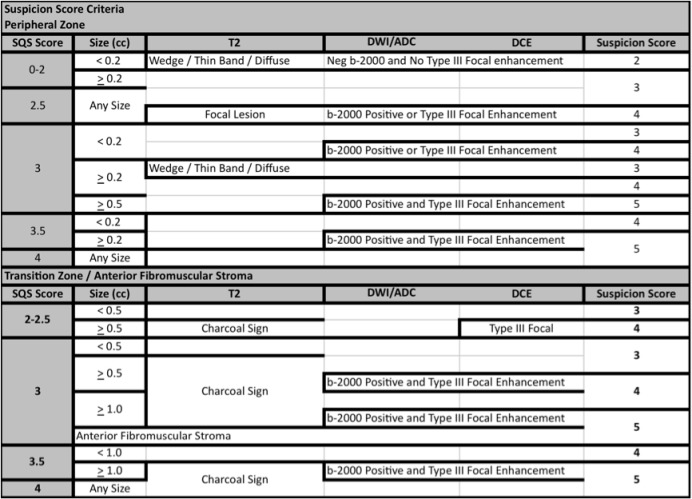
SQS Scoring System, Step 2: Calculate Overall SQS suspicion score. Select PZ or TZ then use the raw score to select the initial level of suspicion. Next move left to right taking into account the T2, ADC, DCE findings. You must meet all the criteria on each line to obtain the overall suspicion score on the right side of the table; otherwise use the lower score.

**Fig 5:**
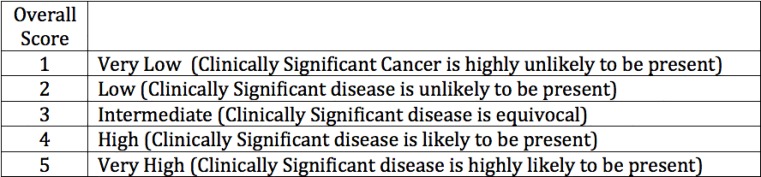
5-point scale: Overall SQS Score and PI-RADS use the same terminology for grading.

Alternative scoring systems have been developed. Herein we report our initial experience using a non-weighted risk stratification for each of the three sequences obtained in a Simplified Qualitative System (SQS) score. The SQS scoring system was adapted from the initial NIH scoring system, which was based on a Boolean approach, in which each sequence was interpreted as being positive or negative and the total number of positive sequences resulted in a suspicion score [9]. There are two steps in calculating the SQS score. In the first step we calculate the SQS Raw score **(Fig 3)**. The raw score represents an initial assessment of risk with each sequence obtaining a score of positive, negative or mild. The next step is to refine the score using the Raw Score, Size, T2 morphology, DWI (high b-value), and DCE (Type III focal enhancement) to increase or decrease the probability of a lesion being positive and harboring clinically significant disease **(Fig 4)**. The SQS work flow was developed to be used during the interpretation of the MRI. No modeling was used to determine impact of individual sequences. Both PI-RADS and SQS use the same language for reporting the probability of clinically significant disease based on a 5 point likert scale **(Fig 5)**. Both systems incorporate size, the PIRADS system sets the threshold at >1.5 cm to determine risk, and the SQS score uses tumor volume thresholds (0.2cm^3^ and 0.5 cm^3^) set forth by Epstein [10].

**Fig 6:**
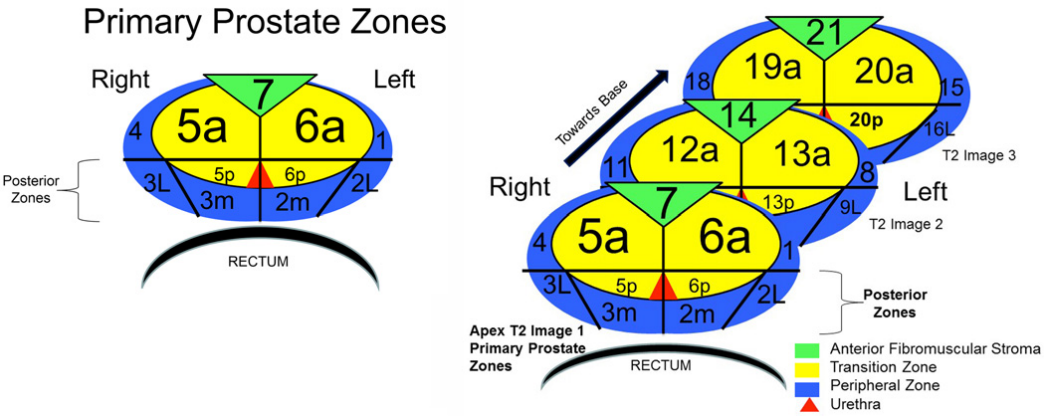
Zonal Anatomy.

Thorough evaluation and staging of the prostate with mpMRI requires a universally accepted scoring system and a clear way to convey the location of the lesion to the physician. PI-RADS describes lesions based on anatomical “zones,” at the levels of the apex, mid and base of the prostate which can be difficult to translate for targeting purposes, because no definite slice number is reported. The SQS, however, reports similar zonal anatomy **(Fig 6)** as well as axial slice numbers, allowing for seamless integration with current fusion biopsy platforms. This study seeks to report our initial experience using our SQS score and to report the impact of the new PI-RADS for their ability to predict prostate cancer using a MR/US fusion guided biopsy approach.

## Methods

### Patients and Data Collection

Patients were enrolled in the IRB-approved (11-322a) phase III prospective trial (National Clinical Trial ID 01566045) at the North Shore LIJ Health System **(S1, S2 and S3 Files)**. Enrollment began in February 2012 and data was analyzed through November 2014. A total of 1060 patients underwent a prostate MRI during the study period. Only those patients with suspicion of CaP (elevated PSA and/or an abnormal DRE) were referred for MRI. Of patients referred for imaging, the PSA range was 0.06 to 80 ng/ml, with a median 6.50 ng/ml (IQR [4.60 to 9.00]). The PCPT HG risk calculator 2.0 was used to estimate the incidence of HG disease for the entire cohort. 506 MRI’s were positive for one or more suspicious lesion(s). 341 patients with no prior history of prostate cancer were referred by their primary urologist for enrollment into the trial, in which the inclusion criteria was an MRI visible lesion on mp-MRI of the prostate. 29 patients were excluded due to technical failure and or they could not tolerate the biopsy with local anesthesia. Patients with suspicious findings on MRI were then offered enrollment in the trial for MR/TRUS fusion-guided biopsy of suspicious lesions in addition to the standard of care (12-core biopsy) performed at the same setting (protocol biopsy).

Demographic, clinical, imaging and histopathologic data were prospectively collected in a secure study database and retrospectively analyzed. The patient-based analysis included biopsy data from the protocol biopsy. The lesion-based analysis only included targeted biopsy results of MRI visible lesions. Epstein's Criteria were used to define clinically significant CaP: any Gleason pattern ≥ 4, or Gleason 3+3 disease with core length ≥ 50% and/or > two cores positive on the standard 12-core TRUS-guided biopsies. Clinically significant CaP on fusion biopsy was defined as any Gleason pattern 7–10 and/or Gleason 6 disease and MRI visible lesion >0.5 cm^3^, which was proposed in the recent update to PI-RADS. [8, 10]

### Imaging Technique and Analysis

**Fig 7:**
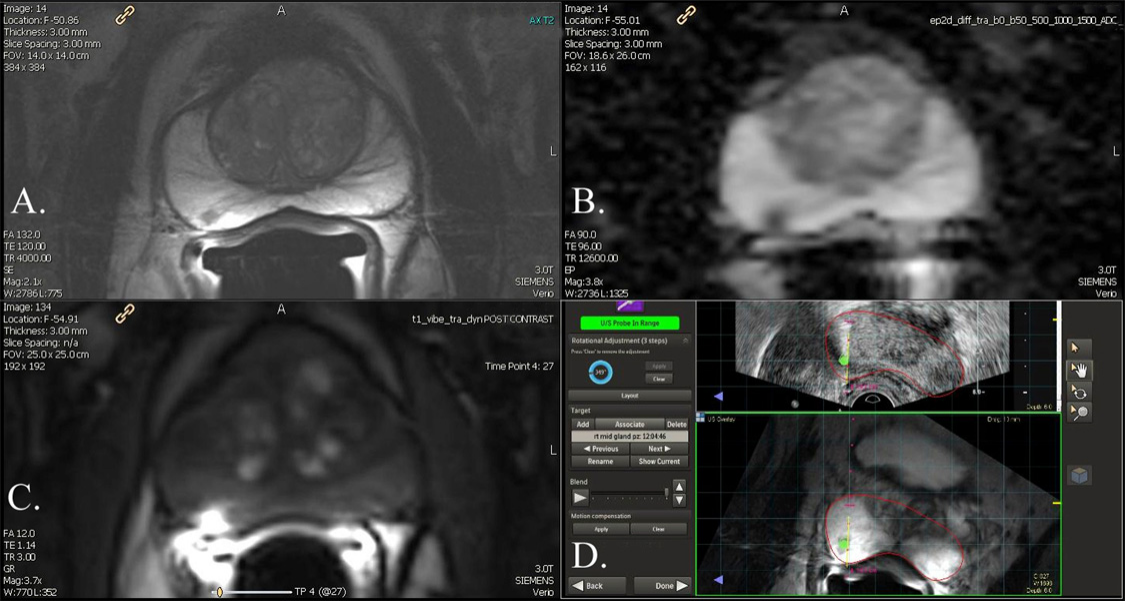
Prostate mpMRI. A. T2 sequence with a low signal intensity in Zone 3L. B. DWI with corresponding ADC restriction C. DCE with early arterial uptake. D. MR US fusion directed biopsy detected Gleason 3+4, 2.5 mm. Please see supplemental report regarding specific lesion based reporting.

All enrolled patients underwent mpMRI (3T Verio, Siemens, Germany ®) of the prostate obtained with a 16-channel cardiac coil (Sense, Invivo ®) on the anterior pelvis and an endorectal coil (BPX-30, Medrad, Pittsburgh, PA ®) filled with PFC-770 (3M, St. Paul, MN ®). Sequences obtained included tri-planar T2-weighted, diffusion-weighted imaging (DWI) (b-values 0, 500, 1000, and 1500) and a separate b-2000 sequence and dynamic contrast enhanced (DCE), **(S1 Table).** A representative image is seen in **Fig 7**. All mpMRI were read in consensus by three experienced GU radiologists (AR, RV, EBL), who assigned risk scores prospectively using the SQS score and PI-RADS v1. The use of consensus reads was implemented at the beginning of the MR fusion biopsy program in step with current recommendations for optimizing prostate MRI interpretations[11]. The PI-RADS v2 score was calculated from existing ‘granular’ data points which were available in the database using a combination of SQS findings and PI-RADS v1.

For example, a lesion **(Fig 7)** that measures 7 x 6 x 5 mm (0.11 cc), with a focal T2 lesion, mild restriction on ADC maps, negative b-2000, early arterial enhancement, and no EPE would have an initial raw score of 2.5 **(Fig 3)**. The raw score can range from 0 to 4. The second step improves risk stratification by taking into account lesion volume, T2 morphology, DWI (b-2000), and DCE (Type III focal enhancement) to determine an overall SQS suspicion score **(Fig 4)**. In this example from the supplemental report **(S4 File)** the SQS overall suspicion score for this lesion would be 4 **(Fig 4)**. In summary, we first stratify by qualitative findings on the MR sequences then use detailed criteria to upgrade or downgrade a lesions suspicion based on the second step **(Figs 3 and 4)**.

All lesions were reported in a standard fashion using zonal anatomy that begins at the apex slice and progresses towards the base **(Fig 6)**. The TZ and PZ are divided into anterior and posterior regions similar to the PI-RADS zonal anatomy. The posterior zone is delineated by an area posterior to a transverse line drawn through the urethra and no further than 1.5 to 2 cm from the posterior border of the prostate, which correlates with the length of a standard biopsy needle depth of penetration. The posterior zone corresponds to the PZ (2 and 3 lateral (L), 2 and 3 medial (M)) and the TZ (5 and 6 posterior (P)). This zonal anatomy allows the urologist to review, confirm, and target specific areas on MR US fusion biopsy systems.

### Biopsy Protocol

MR images were processed on a Dynacad workstation (Invivo, Gainesville, FL ®). MR/TRUS image fusion was performed using UroNav ® software in conjunction with an IU-22 (Philips Health Care, Best Netherlands) end-fire ultrasound probe. During biopsies of the lesions, one core was obtained in the axial and sagittal planes for a total of two cores per lesion. Following targeted biopsy, a standard 12-core TRUS-biopsy was performed after the MR-fusion system was disabled.

### Statistical Analysis

This is a descriptive study taken from the data of National Clinical Trial ID 01566045, which was originally powered to detect the difference between a standard ultrasound guided biopsy vs. MR US fusion guided biopsy for the detection of cancer at a NIH suspicion score of low, moderate and high on mpMRI of the prostate. A separate power calculation was not performed prospectively for this descriptive study. However, we retrospectively calculated the power based on computing the difference between the two means of the each scoring system 3.7 (SQS) and 3.9 (PIRADS), a standard deviation of 0.8, an alpha set at 5% using the 312 patients, we were able to achieve a 99.3% statistical power. All tests were two-sided. Cancer detection rates (CDR) and the CDR for clinically significant disease were compared across each score using the Pearson chi-squared test for SQS and PI-RADS. Negative predictive value (NPV), positive predictive value (PPV), sensitivity and specificity were calculated at all possible cut-points for both mpMRI scoring systems with respect to their associations with a biopsy-proven diagnosis of prostate cancer. Receiver-operating characteristic curves were mapped and compared for each scoring system (Analyse-it ® v3.90.1, Analyse-it Software Ltd, Leeds, UK). Lesion-based (fusion biopsy only) and patient-based (fusion and standard 12-core biopsies) analyses were performed with respect to the detection of cancer and the detection of clinically significant disease. The patient-based analysis was performed using the index lesion (highest risk lesion) to define overall risk for each of the scoring systems. The AUCs were calculated in a paired design and a DeLong Clarke-Pearson method was used to compare the corresponding curves.

## Results

A total of 312 patients **(S5 File)** underwent biopsy of 452 lesions **(S6 File)**. Median patient age was 65.1 years (IQR 60.3–70.3) and median PSA was 7.30ng/mL (IQR 4.99–11.4). 86.2% of digital rectal exams were negative. The overall (fusion and 12-core) cancer detection rate was 64.7% (202 /312), of which 17% (35/202) of cancers were detected solely on the 12-core biopsy and 16% (33/202) on fusion biopsy alone. In patients where one of the two approaches missed cancer, fusion biopsy detected 81.8% (27/33) of clinically significant prostate cancer compared to the 12 core biopsy which detected 31.4% (11/35) of clinically significant prostate cancer. An average of 1.4 MRI visible lesions per patient (452 lesions total; 1–4) were biopsied, 246 targets were noted to be negative (54.4%) and 206 were positive (45.6%). Both scoring systems demonstrated an increase in cancer detection rate with an increasing score on both a per-patient- and per-lesion-basis (all *p<0*.*0001*, **Table 1**).

**Table 1:** Cancer detection rates for prostate cancer and clinically significant prostate cancer stratified by scoring system.

Patient-level analysis				Lesion-level analysis		
**SQS Suspicion Score**	**Total**	**Cancer Detection Rate**	**Clinically Significant CaP**	**Total**	**Cancer Detection Rate**	**Clinically Significant CaP**
2	12	25.0%	16.7%	24	8.3%	0.0%
3	135	44.4%	25.9%	231	23.8%	14.3%
4	115	77.4%	70.4%	145	67.6%	57.9%
5	50	100.0%	96.0%	52	98.1%	96.2%
**PI-RADS**	**Total**	**Cancer Detection Rate**	**Clinically Significant CaP**	**Total**	**Cancer Detection Rate**	**Clinically Significant CaP**
1	5	0.0%	0.0%	*7*	14.3%	14.3%
2	28	25.0%	21.4%	*49*	22.4%	10.2%
3	23	39.1%	13.0%	*47*	17.0%	6.4%
4	195	67.7%	54.4%	*283*	46.3%	36.7%
5	61	88.5%	83.6%	*66*	83.3%	81.8%
Total	312	64.7%	53.2%	*452*	45.6%	36.9%

Among patients with cancers, clinically significant disease was found in 82.2% (166/202) of patients. PI-RADS and SQS exhibited an increasing cancer detection rate for clinically significant cancer with an increasing score (*p<0*.*0001*; **Table 1**).

**Fig 8:**
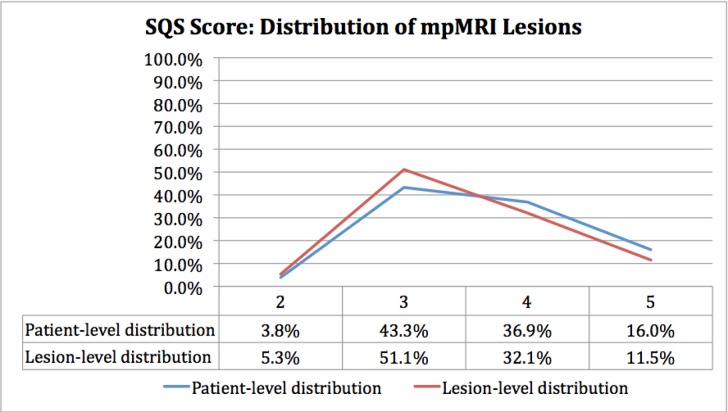
Distribution of lesions reported on mpMRI stratified by SQS Score.

**Fig 9:**
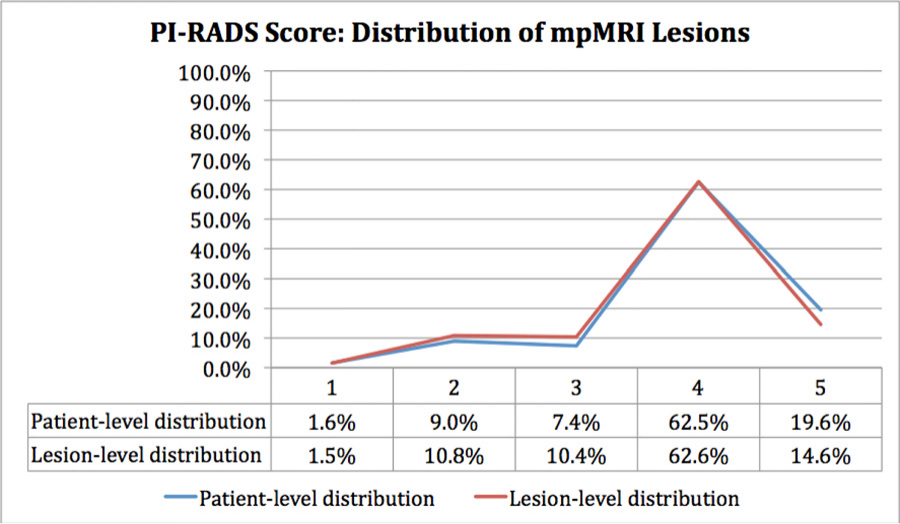
Distribution of lesions on mpMRI stratified by PI-RADS Score.

**Fig 10:**
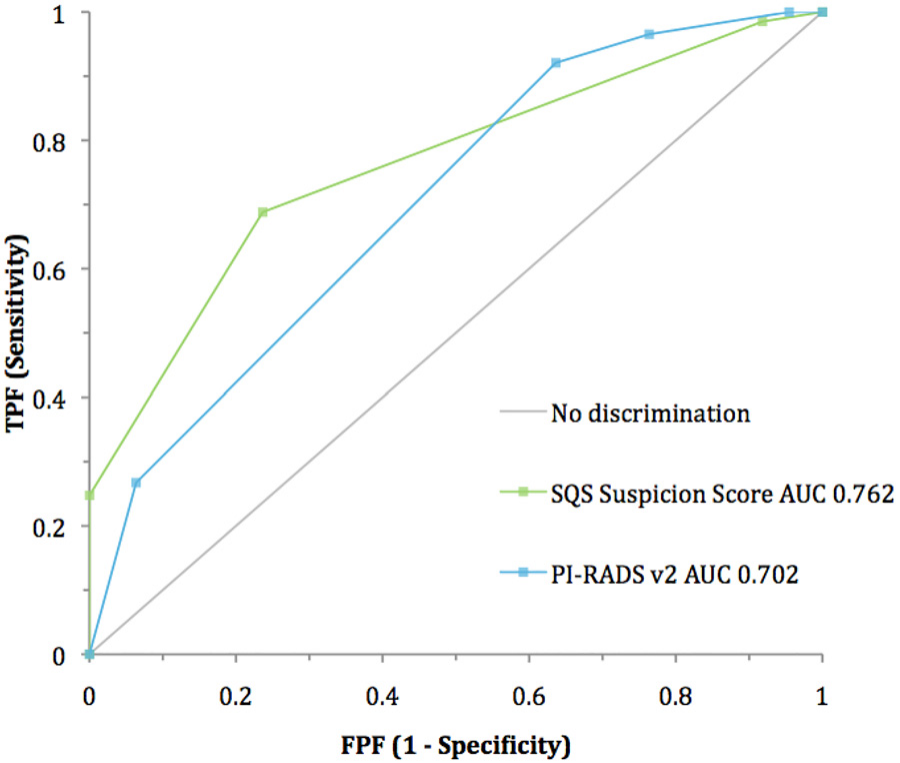
ROC curve analysis: Patient-based.

**Fig 11:**
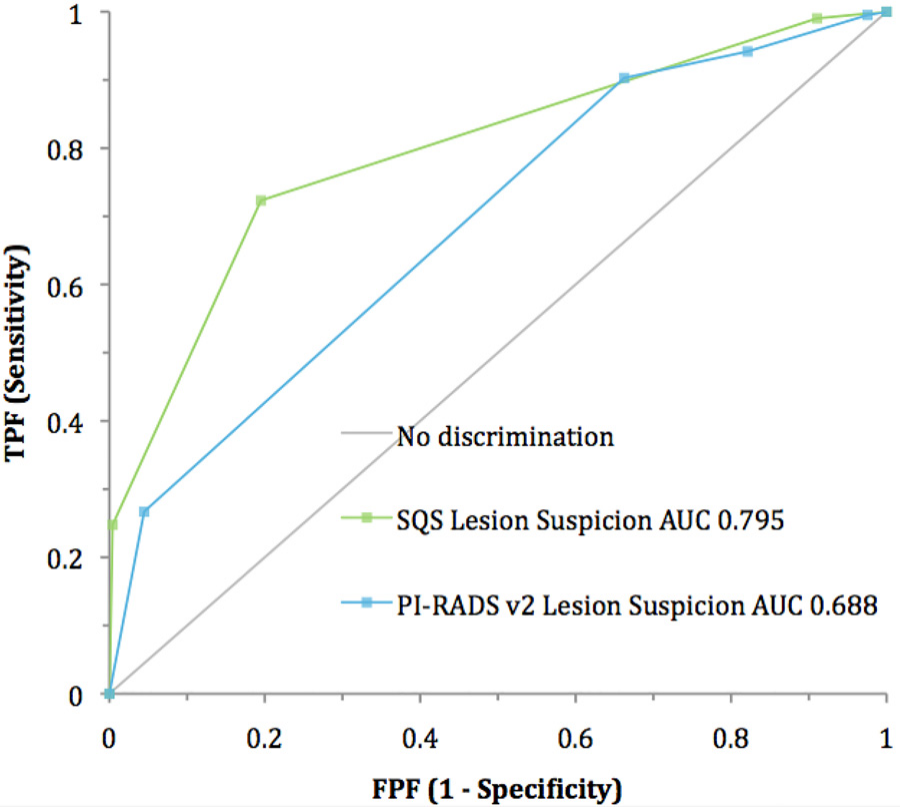
ROC curve analysis: Lesion-based.

Analysis of the distribution of positive cases by lesion score demonstrates a bell-shaped curve for the SQS score **(Fig 8)** while the PI-RADS score was skewed toward higher scores **(Fig 9)**. Receiver-operating characteristic (ROC) curves for both scoring systems demonstrated areas under the curve (AUC) that were significantly greater than non-discrimination (*p <0*.*0001*) for patient-based **(Fig 10)** and lesion-based analysis **(Fig 11)**. The SQS outperformed the PI-RADS score on a per-patient and per-lesion basis with respect to detection of prostate cancer and clinically significant prostate cancer (p < 0.0001). The calculated AUC with respect to predicting prostate cancer for PI-RADS was 0.702 (95% CI 0.65 to 0.75) and 0.688 (95% CI 0.65 to 0.73) on a per-patient and per-lesion basis. In comparison, AUC for SQS score was 0.762 (95% CI 0.72 to 0.81) and 0.795 (95% CI 0.76 to 0.83) on a per-patient and per-lesion basis, respectively. Similarly, in our evaluation of clinically significant prostate cancers, the PI-RADS score demonstrated an AUC of 0.705 (95% CI 0.66 to 0.75) and 0.725 (95% CI 0.69 to 0.76) compared to the SQS score of 0.799 (95% CI 0.75 to 0.84) and 0.829 (95% CI 0.79 to 0.87) on a per-patient and per-lesion basis, respectively (p<0.0001). PPV, NPV, Sensitivity and specificity of each system are listed in **Tables 2 and 3.**

**Table 2:** Positive Predictive Value, Negative Predictive Value, Sensitivity and Specificity at each cut point in each scoring system for the detection of clinically significant prostate cancer: Patient-level analysis.

Clinically Sig. Cancer Detection: Patient-level				
**SQS**	**Sen**	**Spec**	**PPV**	**NPV**
**2**	98.8%	6.8%	54.7%	83.3%
**95%CI**	0.96 to 1.00	0.04 to 0.12	0.54 to 0.56	0.53 to 0.96
**3**	77.7%	75.3%	78.2%	74.8%
**95%CI**	0.71 to 0.83	0.68 to 0.81	0.73 to 0.83	0.69 to 0.80
**4**	28.9%	98.6%	96.0%	55.0%
**95%CI**	0.23 to 0.36	0.95 to 1.00	0.86 to 0.99	0.53 to 0.57
**5**	0.0%	100.0%	-	46.8%
**95%CI**	-to-	-to-	-to-	-to-
**PI-RADS**	**Sen**	**Spec**	**PPV**	**NPV**
**1**	100.0%	3.4%	54.1%	100.0%
**95%CI**	0.98 to 1.000	0.02 to 0.08	0.94 to 1.08	0.00 to 0.67
**2**	96.4%	18.5%	57.3%	81.8%
**95%CI**	0.92 to 0.98	0.13 to 0.26	0.55 to 0.59	0.66 to 0.91
**3**	94.6%	32.2%	61.3%	83.9%
**95%CI**	0.90 to 0.97	0.25 to 0.40	0.59 to 0.64	0.73 to 0.91
**4**	30.7%	93.2%	83.6%	54.2%
**95%CI**	0.24 to 0.38	0.88 to 0.96	0.73 to 0.91	0.51 to 0.57
**5**	0.0%	100.0%	-	46.8%
**95%CI**	-to-	-to-	-to-	-to-

**Table 3:** Positive Predictive Value, Negative Predictive Value, Sensitivity and Specificity at each cut point in each scoring system for the detection of clinically significant prostate cancer: Lesion-level analysis.

Clinically Sig Cancer Detected: Lesion Level				
**SQS**	**Sen**	**Spec**	**PPV**	**NPV**
**2**	100.0%	8.4%	39.0%	100.0%
**95%CI**	0.98 to 1.00	0.06 to 0.12	0.38 to 0.40	- to -
**3**	80.2%	77.9%	68.0%	87.1%
**95%CI**	0.74 to 0.86	0.72 to 0.82	0.62 to 0.73	0.83 to 0.90
**4**	29.9%	99.3%	96.2%	70.8%
**95%CI**	0.24 to 0.37	0.97 to 1.00	0.86 to 0.99	0.69 to 0.73
**5**	0.0%	100.0%	-	63.1%
**95%CI**	-to-	-to-	-to-	-to-
**PI-RADS**	**Sen**	**Spec**	**PPV**	**NPV**
**1**	99.4%	2.1%	37.3%	85.7%
**95%CI**	0.97 to 1.00	0.01 to 0.05	0.37 to 0.38	0.42 to 0.98
**2**	96.4%	17.5%	40.7%	89.3%
**95%CI**	0.92 to 0.98	0.14 to 0.22	0.39 to 0.42	0.79 to 0.95
**3**	94.6%	33.0%	45.3%	91.3%
**95%CI**	0.90 to 0.97	0.27 to 0.38	0.43 to 0.47	0.84 to 0.95
**4**	32.3%	95.8%	81.8%	70.7%
**95%CI**	0.26 to 0.40	0.93 to 0.98	0.71 to 0.89	0.68 to 0.73
**5**	0.0%	100.0%	-	63.1%
**95%CI**	-to-	-to-	-to-	-to-

A Pearson chi-squared analysis of cancer detection rate by lesion volume demonstrated that MRI lesion volume impacted cancer detection rate (*p<0*.*0001*). Lesions <0.2cm^3^ were positive in 39.5% (70/177) of cases, lesions 0.2 to < 0.5cm^3^ were positive in 45.2% (57/126) of cases, lesions 0.5 to <1cm^3^ were positive in 43.3% (29/67) of cases, and lesions > 1cm^3^ were positive in 61.0% (50/82).

## Discussion

In the wake of the United States Preventative Services Task Force’s issuance of a Grade D recommendation against prostate cancer screening with PSA [12], a number of researchers have focused their efforts on identifying ways to improve pre-treatment risk stratification, including the use of urinary markers [13], genetic analysis [14], and imaging to better characterize the biology of the disease [6, 9, 15, 16]. Urinary markers such as PCA3 have demonstrated sensitivity of 68.4% and specificity of 58.3% (cutoff score of 35), while genetic markers such as TMPRSS2-ERG have demonstrated a sensitivity of 24.3% and specificity of 93.2% [17]. In our study, we demonstrated that mpMRI is a useful diagnostic tool in detecting clinically significant prostate cancer, with a sensitivity and specificity as high as 80.2% and 77.9% (SQS score of 3), respectively, for individual lesions appreciated on mpMRI. The unique difference that separates mpMRI from the other secondary markers is that it not only detects the presence of cancer but can also be used to direct biopsies, thereby overcoming the inherent limitations associated with the standard 12 core ultrasound guided biopsy and genetic markers. While mpMRI offers the potential benefit of improved risk stratification, it nonetheless requires the universal adoption of a standardized scoring schema to provide an objective framework in guiding image interpretation across centers.

Standardized scoring schemas have been described in other organ systems (Breast, Thyroid, and Lung) [18–20]. The common goals of each system are to guide interpretation in order to minimize subjectivity, standardize communication and facilitate reproducibility and comparability. Similarly, the PI-RADS and SQS score were created to fill the void of standardized data reporting in prostate imaging. As our understanding evolves and criteria change, it is important to maintain datasets that are able to test and validate proposed changes to the scoring systems. We are not intending to promote one scoring system over the other. The aim of this paper was to illustrate the strengths and weaknesses of the scoring systems and the added advantage of recording ‘granular’ (sequence specific data including lesion morphology and enhancement curves) data in a way that these analyses can be made in the future. If one converts to PIRADS v2 only some sequence specific data can be lost and limit one’s ability to make adjustments to forthcoming changes in scoring systems.

Mapping the location of identified lesions is a key function of a reporting standard. The SQS and PI-RAD system rely on the well-known anatomical zones for visual reporting that may aid in localizing suspicious areas while performing MR-TRUS fusion biopsies. The differences are that PI-RADS uses generalized locations with respect to the apex, mid and base to describe the location of the lesion. The SQS zonal anatomy was developed prior to the PI-RADS v2 update and reports T2 axial slice numbers starting from the apex and progressing cephalad, slice by slice, allowing us to account for the variability at the base. The SQS reports all lesions based on the primary prostate zones (Peripheral, transitional, central zones) and correlates them with the axial slice numbers on the T2 weighted images. (Supplemental Sample Report).

Assuming a normal distribution of patients in a population, one would expect a scoring system to have a Gaussian distribution in which the majority of patients would have scores in the middle of the scale. The population studied here was approximately normal with a broad range of risk levels. According to the PCPT HG risk calculator 2.0, the estimated the incidence of HG (Gleason > = 7) prostate cancer was 125.7/1060 cases [21]. In our series there were 150/312 patients that were diagnosed with HG disease. This is approximately 24 more cases of HG disease than one would have obtained if a standard 12 core biopsy was done in all 1060 patients. Moreover, this figure does not include the 165 patients who had a positive MRI who were not referred to the trial. The positive MRI rate in this study was 56.4% (241/427), 41.9% (265/633), and 47.7% (506/1060) for patients who were biopsy-naïve, had a prior negative biopsy, and for the entire cohort, respectively. The question then arises, what is the optimal threshold to report an MRI positive with a visible lesion? Unfortunately, this analysis would be beyond the scope of this publication. However, when comparing to the large systematic review the biopsy naïve and prior negative biopsy positive MRI rates were reported to be 66% and 69% respectively [22]. It is difficult to draw conclusion from a large review, but we utilized the PCPT HG calculator to provide an overall estimate of high grade disease to determine if our threshold was set too high for reporting an MRI as being positive.

The PI-RADS scoring system was skewed toward higher scores with 82.1% of positive cases being scored as a 4 or 5 at the patient level. The SQS scoring system is less strongly skewed and offers a more balanced distribution of scores, with only 52.9% of cases scored as a 4 or 5, and 47.1% scored as a 2 or 3. The nature of the SQS scoring system allows it to take advantage of the entire scale to differentiate among lesions with varying degrees of suspicion for cancer with a demonstrated performance advantage. The PI-RADS scoring system, on the other hand, may potentially weaken risk stratification by narrowing the 5-point ordinal scale to two numbers.

The SQS scoring system also outperformed PI-RADS in the detection of all cancers and of clinically significant cancers on a per-patient and per-lesion basis. While both scoring systems were found to have acceptable sensitivity and specificity, SQS was more accurate, demonstrating consistently higher AUCs on both a per-patient and per-lesion basis. Finally, it is important to note that the PI-RADS recommendations were not based on an analysis of a large data set, but rather were a result of expert opinion and may need further refinement. There is currently discussion on PIRADS v3. As stated in the document from the ACR, PI-RADS is not meant to be used as a tool to determine when to perform a biopsy or not. Rather, these scoring systems should be used in clinical context to improve risk stratification and help a physician make a clinical decision.

Some limitations of this study are that the PIRADS v2 was performed using data points acquired prior to the publication of the PIRADS v2. However, by their nature, imaging studies are amenable to such retrospective analyses. When the study was initially conducted, only PIRADS v1 was available; however, we felt it was more appropriate to utilize the newest PI-RADS recommended version upon its release. Another limitation is that fusion biopsy is a technically complex technique with a steep learning curve and carries the potential to miss the cancer observed on MRI. This series only included patients with MRI visible lesions enrolled in the trial. As such, the false negative rate of imaging may be artificially inflated due to the possibility of missed targets. Also, the entire cohort (1060) was not biopsied as some patients were not a part of the fusion biopsy trial and were referred to our institution for imaging only. These data are being acquired through an ongoing research project, but is beyond the scope of this article. The use of an isolated high b-2000 DW image may not be feasible for community-based practices due to time constraints. However, as magnet quality improves, acquisition time has decreased and the PIRADS v2 does recommend the possible benefits of a single high b-value 1400–2000 sec/mm^2^ DW imaging sequence [8]. Finally, our use of consensus reads by three trained GU Radiologists, a single experienced GU pathologist reviewing all specimens, and a single physician performing biopsies *and* reviewing images, may not reflect routine practice in the general community.

## Conclusions

mpMRI is an accurate tool that has the potential to enhance pre-biopsy risk stratification in patients with suspicion of prostate cancer. The PI-RADS and SQS scoring systems both have acceptable sensitivity and specificity in detecting prostate cancer. SQS offered a more normal distribution of scores, and produced a higher AUC on a per-patient and per-lesion basis compared to PI-RADS. These findings suggest that SQS offers greater diagnostic accuracy in patients imaged with mpMRI for prostate cancer. As our understanding of prostate MRI evolves it is important to continue to record clinical data prospectively at a granular level which would allow the validation and analysis of modifications to the scoring systems.

## Supporting Information

S1 File IRB-approved protocol for (11-322a) phase III prospective trial (National Clinical Trial ID 01566045). (DOCX)

S2 File IRB approval letter. (PDF)

S3 File Transparent Reporting of Evaluations with Nonrandomized Designs (TREND) trial checklist. (PDF)

S4 File Sample SQS-based MRI report with anatomic detail and axial slice number. (DOCX)

S5 File Patient-level data set. (XLSX)

S6 File Lesion-level data set. (XLSX)

S1 Table MRI settings. (DOCX)
