# Supplementary material for: Comparison of Multiparametric MRI Scoring Systems and the Impact on Cancer Detection in Patients Undergoing MR US Fusion Guided Prostate Biopsies
Source: PLoS One. 2015 Nov 25;10(11):e0143404. doi: 10.1371/journal.pone.0143404 (PMC4659614; doi:10.1371/journal.pone.0143404)
Supplement: S1 File — (DOCX) [file pone.0143404.s001.docx]

**MR/TRUS FUSION GUIDED PROSTATE BIOPSY– AN IMPROVED WAY TO DETECT AND QUANTIFY PROSTATE CANCER.**

**A PHASE 3 STUDY**

Principal Investigator: Ardeshir R. Rastinehad, DO

Associate Investigator: David N. Siegel MD

Associate Investigator: Eran Ben-levi MD

Associate Investigator: Louis R. Kavoussi MD

Associate Investigator: Igor Lobko

Contact: Ardeshir R. Rastinehad, DO

The Arthur Smith Institute for Urology

450 Lakeville Road

New Hyde Park, NY 11040

Phone: 516-734-8500

Fax: 516-734-8535

Pager ID - 91281

FDA information for device -

12/19/2005

Product Name: ABARIS

Common name: Computer assisted, image-guided surgery system

Classification Name: Computed tomography X-Ray system Class II as described: 21CFR 892.1750

Product code: JAK 510(k) K053610

**INTRODUCTION/BACKGROUND**

The efficacy of targeting lesions for surgery, CT-guided, or ultrasound-guided biopsy, or ablation, currently may be limited by the visibility of a target during the procedure. The successful outcome of surgical intervention depends upon accurate device placement, which may be very challenging in certain settings, such as when a kidney tumor only is visible for a brief moment during the transient arterial phase of a contrast injection, and soon disappearing on dynamic imaging.

Historically, prostate cancer was diagnosed by digitally guided trans-rectal prostate biopsies. ^[[1]](#endnote-1),^ ^[[2]](#endnote-2)^ However, with the advent of PSA screening and improvements in ultrasonography, trans-rectal ultrasound guided prostate biopsy have become the standard of care to screen and diagnose localized prostate cancer.^[[3]](#endnote-3),^ ^[[4]](#endnote-4)^ These biopsies are performed following a random sextant scheme to sample the prostate. In order to improve the diagnostic yield of the prostate biopsy technique, the number of cores obtained during a prostate biopsy has increased.^[[5]](#endnote-5)^ In fact, an extended (standard) 12-14 core prostate biopsy is now common practice, detecting cancer in 27%^[[6]](#endnote-6)^ to 44%^[[7]](#endnote-7)^ of patients.

Initially, prostate MR imaging was not considered for routine clinical practice.^[[8]](#endnote-8)^ However, the addition of an endorectal-coil probe and a 3 Tesla magnet has improved its diagnostic utility dramatically.^[[9]](#endnote-9),^ ^[[10]](#endnote-10)^  MR-guided prostate biopsies have been performed in the MR suite.^[[11]](#endnote-11),^ ^[[12]](#endnote-12)^ Known as “in gantry” biopsies, these biopsies are difficult, time-consuming, and require specialized equipment, which increases the cost significantly.

Therefore, we have developed a unique alternative approach “Out-of-gantry MR/TRUS fusion guided biopsy”. We perform focal prostate procedures, such as a prostate biopsy, with the ease and familiarity of the real-time trans-rectal ultrasound that urologists use today. This is accomplished by incorporating MR images for guidance, which we believe may have a significant impact on the diagnosis and treatment of prostate cancer.

To meet this challenge, a custom platform has been constructed (UroNav) that fuses real-time TRUS images with a previously obtained prostate MRI, combined with an electromagnetic tracking system. The urologist then performs directed prostate biopsies at MR-identified targets in addition to the standard 12-14 core biopsies. The technical aspects of this platform have been previously described in pre-clinical and animal models,^[[13]](#endnote-13),^ ^[[14]](#endnote-14)^ and we have reported the results of our first 101 patients that have undergone both conventional standard biopsy and MRI/ultrasound fusion-targeted biopsy of the prostate using our platform.^[[15]](#endnote-15)^ Currently, the unpublished series from the National Cancer Institute is greater than 400 patients using the UroNav system.

The UroNav system utilizes an electromagnetic tracking system (Philips Healthcare, Best, Netherlands) that gives position and orientation information that our custom software ( and software upgrades) then displays on selected registered images. A weak tetrahedral magnetic field generator is placed near the working space of the patient and the Faraday Effect induces a very small passive current in the sensor coil (embedded within the prostate needle biopsy guide ) based upon exact distance from a magnet. The alternating current magnetic field is about 12 kHz. The software (and software upgrades) can combine the distances from each magnet to give exact location, similar to how a global positioning system (GPS) localizes a car with multiple satellites. This system (UroNav) is only a software modification on the FDA approved 510(K), ABARIS system, 2009, for image guided surgery.

Electromagnetic tracking of instruments in interventional radiology/urology can provide real-time display of position and orientation within previously-acquired 3D imaging data sets. This may facilitate use of pre-procedural spatial or anatomic information during interventional/urological procedures. This registration joins imaging space to magnetic space of the patient. The patient is also imaged in real time with the same or a different modality. Tracking may enable accurate and precise navigation of trans-rectal ultrasound guided biopsies of the prostate. Accuracy, precision and increased quantification of a patient’s cancer could translate into improved outcomes and patient care.

Although standard image-guided biopsy and ablation uses morphologic imaging, there may be a role for navigation with functional or metabolic imaging. Both are possible with electromagnetic tracking registration of pre-procedural imaging. Dynamic MR datasets to guide biopsy locations may facilitate the spatial analysis of cancer therapies. Accurate biopsy targeting to metabolically active regions may decrease effect of spatial heterogeneity of tumors. Sequential biopsies may be performed to analyze the genomics and proteomics of tumor growth and response to therapy.

Pre-Protocol MR Imaging:

Patients will have undergone multiparametric imaging using a 3.0 T MRI scanner combined with a 6-channel cardiac surface coil (or equivalent) positioned over the pelvis and an endorectal coil (BPX-30, Medrad, Pittsburg, PA, USA) or comparable type of endorectal coil. Tri-planar T2-weighted, axial dynamic contrast-enhanced, axial diffusion weighted imaging with ADC mapping MR imaging sequences, and/or three-dimensional (3D) point resolved spatially localized spectroscopy, are conducted according to protocol. Details of these imaging sequences have been described previously.^11,21^ The imaging protocol for the prostate will be in adherence to the current consensus panel from the National Institutes of Health (NIH), the Molecular Imaging Program (MIP) directed by Dr. Peter Choyke and/or his designee, this will allow the protocol to stay current with advances in the field and not deprive patients of imaging advancements while in accrual.

The criterion for a positive lesion on T2-weighted and diffusion weighted imaging is a well-circumscribed, round-ellipsoid low-signal-intensity lesion.^11^ A positive lesion on dynamic contrast-enhanced imaging is a presence of foci showing early and intense enhancement and rapid washout. A positive lesion on spectroscopy is an area where the choline-citrate ratio is 3 or more standard deviations above the mean healthy value.^21^

Radiologists identify and grade the lesions suspicious for cancer according to the number of MR imaging sequences suspicious for cancer: low (<2), moderate (3) and high suspicion (4 sequences). The radiologists are blinded to pre-imaging serum PSA values, prior biopsy status and previous histopathologic findings. Each MRI sequence will be evaluated independently and separately. The images obtained here at the NSHS LIJ Health system will be read by our imaging team, Dr. Ben-levi or his designee. After obtaining the consent of the patient, a consult with the NIH/MIP (Dr. Choyke or his designee) maybe called to review the films in accordance with the NIH consult policy and HIPPA compliance.

**OBJECTIVES:**

1. To determine if targeted (MR / US fusion biopsy) plus conventional biopsy is superior to conventional biopsy alone in diagnosing subjects with prostate cancer.

**EXPERIMENTAL DESIGN AND METHODS**

This study will consist of comparison the standard of care (TRUS guided prostate biopsy) with the protocol biopsy which consists of a TRUS guided prostate biopsy and a MR/US fusion tracked prostate biopsy. Each patient will act as their own control.

Step 1: REGISTRATION, SEGMENTATION and TARGET ACQUISITION

The imaging study series of interest will be transferred in standard DICOM format to the UroNav platform (hardware and software). The magnetic field generator will be placed in close proximity to the patient near the working space. The patient will then be registered to the pre-operative imaging data set by performing the prostate 3D US image acquisition allowing for volume rendering and registration. The tracking coil is placed within the standard TRUS prostate biopsy needle guide (Philips Healthcare, Best, Netherlands).

Step 2: BIOPSY (Standard 12 core biopsy and MR/US guided fusion prostate biopsy)

Patients will first receive a standard of care 12-core TRUS sextant biopsy of the medial and lateral margins of the right and left apex, mid-gland and base of the prostate. The patients will receive antibiotic prophylaxis as per the American Urologic Association guidelines. For the 12-core TRUS biopsy, the operator is blinded to the location of suspicious lesions identified on pre-biopsy MRI.

At the same setting, patients then will receive a MRI/US fusion guided biopsy under EM tracking of suspicious lesions identified on MRI. There are approximately 4-6 extra biopsies taken (2 biopsies per suspicious lesion seen on MRI)

**EXPERIMENTAL DESIGN STATISTICAL CONSIDERATIONS AND DATA ANALYSIS**

**Statistical Methods/Sample Size Considerations:**

A previous study of 193 patients conducted at Urology Oncology Branch of National Cancer Institute demonstrates that prostate cancer detection rate increased with suspicion level which is based on the number of MR imaging sequences suspicious for cancer. In both low and moderate suspicion levels, there is little difference in the detection rate between the MR fusion biopsy and standard biopsy. When the suspicion level is high, the detection rate by MR-fusion biopsy is 76%, compared to 65% by the standard biopsy, and 85% by the two biopsy methods combined. Based on these findings, for the planning of a new study, the power is targeted at a detection rate difference between the two biopsy methods for the high suspicion group.

Power calculation is based on comparing detection rate using standard vs. MR-US fusion biopsy for patients with high suspicion for cancer. Assume that the patient population for the future study is similar to the previous study population described above, and the difference in the detection rate between the two biopsy methods is similar and equals 11%. Since both biopsy methods will be applied to each patient, the difference in the detection rate is tested by the McNemar’s chi-squared test. To permit the study to achieve 90% power at the 5% significance level, 240 patients with high suspicion is required.

For low and moderate suspicion, the detection rates between the two biopsy methods are expected to be similar, and the sample size is determined to estimate the detection rate with a desired precision. Based on the previous study, assume that the detection rate by MR fusion following standard biopsy equals 36% and 59% for low and moderate suspicion level, respectively. To have the width of the 95% expected confidence interval equal to ± 5%, 360 and 380 evaluable patients are required for the low and moderate suspicion subgroup, respectively. With the three suspicion groups combined, a total of 980 evaluable patients are required for the new study.

Interim analyses will be performed throughout the study period with a minimum analysis occurring bi-yearly for publication and review of the system performance. (see Interim Analysis Plan Section)

**E. PROTECTION OF HUMAN SUBJECTS**

**Recruitment:**

**Inclusion Criteria**

Patients must fulfill all of the following criteria to be eligible for study admission:

1. All patients must have a pre-operative MRI performed in accordance with our NSHSLIJ/NIH MR prostate imaging guidelines.
2. Age greater than 18 years.
3. No serious concurrent medical illness that would preclude the patient from making a rational informed decision on participation.
4. The ability to understand and willingness to sign a written informed consent form, and to comply with the protocol. If in question, an ethics consult will be obtained.
5. Ability to tolerate conscious sedation (if procedure to be performed with conscious sedation and without general anesthesia).
6. PSA >2.5 or Abnormal digital rectal exam or current recommendations for biopsy from the American Urological Association
7. Pre-biopsy prostate MRI as described above, showing targetable lesions within 3 months of biopsy
8. Able to tolerate a TRUS guided biopsy

**Exclusion Criteria:**

Patients with any of the following will be excluded from study entry:

1. Patients with an altered mental status that precludes understanding or consenting for the biopsy procedure will be excluded from this study.
2. Patients unlikely able to hold reasonably still on a procedure table for the length of the procedure.
3. Inability to hold breath, if procedure will be performed with conscious sedation, and without general anesthesia.
4. Patients with pacemakers or automatic implantable cardiac defibrillators (contraindications to MRI)
5. Patients with uncorrectable coagulopathies.

**RECRUITMENT AND CONSENT PROCEDURES:**

Subjects who meet inclusion will be consented by professionals from the Arthur Smith Institute of Urology of the North Shore-LIJ Health System. Smith Institute surgeons who have appropriate patient populations will be made aware of the research study protocol and procedures, and given an overview of the study through contacts with the Principal Investigator and/or staff of this study. The surgeon will identify potential study participants. If the patient expresses interest in participation, the surgeon will either

1) Obtain informed consent (if they are listed as an investigator on this study), or

2) Provide the patient with the study coordinator’s contact information, or

3) Provide the patient’s contact information to the study coordinator with the patient’s permission, which will be documented in the medical record.

After a discussion about the study with a potential subject, interested parties will be given a copy of the consent form by one of the investigators. The investigator will review and explain the consent form with the potential subject. All information about the study will be provided. Ample time will be given for individuals to ask questions regarding participation and to have questions answered prior to signing the consent form. If so desired, those interested will be given a copy of the consent form to take home so that they may have the opportunity to discuss participation further with family and/or advisors. Only those investigators listed in the study protocol will obtain informed consent. If an individual chooses to enroll, the consent form will be signed before participation begins. Once an individual joins the study and informed consent is obtained, the subject will receive a signed copy of the consent form. The subject may withdraw from the study at any time without repercussions to subsequent care.

The research portion of the study applies to the use of the transrectal ultrasound with GPS tracker and the collection of additional biopsies of the suspicious areas seen on MRI.

Additional recruitment may include press releases from North Shore LIJ health system, posting on websites (North Shore and Non Health System websites), local TV and radio.

All office visits and counseling regarding the diagnosis and screening of prostate cancer is part of the standard procedure for a patient with an elevated PSA and undergoing a prostate biopsy.

**DISCOMFORTS AND RISKS**

The risks and discomforts of the procedure are expected to be minimal when compared to the standard of care the 12 core transrectal ultrasound guided prostate biopsy. The only difference is the additional number of biopsies obtained (approximately 4 more per patients). Previous data has shown that most patients have a mean number of targeted lesions of 2.2 in our series from the NIH. Therefore, one biopsy is taken in the axial and one in the sagittal planes. If under sampling (which is assessed by the physician at the time of the procedure) occurs during the biopsy, an additional biopsy may be taken of the target lesion. There are approximately an additional 4 core biopsies obtained per patient. There is no increased morbidity associated with the additional cores.^[[16]](#endnote-16)^

It is normal to expect some minor bleeding after a prostate biopsy. Blood in the urine, semen and with bowel movements may occur intermittently for a few days and possibly even for a few weeks although unusual. Risks of transrectal prostate biopsies may also include infection, which is more common in men with prostatitis and rarely blood clots cause blockage and difficulty urinating. The collection of the 4 extra biopsies for this study does not increase the risk of the biopsy procedure overall nor does it increase the risk of infection.^16^

**POTENTIAL BENEFITS**

No direct benefits are anticipated for participants. However, there is a theoretical benefit due to increased quantification of prostate cancer may allow improved treatment decisions for the patient and practitioner. It is anticipated that participation in this study will benefit the scientific community and increase scientific knowledge of urologic and/or other diseases. Additionally, knowledge may be gained that could change standard practice and patient counseling regarding the surgical employment of tracking systems during image guided interventions.

**Data Safety Monitoring Board (Adverse Events)**

DSMB is not in placed, but the data will be reviewed by the PI and additional experts (as indicated below during the interim analysis.

**Interim Analysis Plan:**

The safety of study participants—including the monitoring and reporting of any adverse events—as well as the integrity of all data generated from this study will be closely followed by the principal investigator and the research department at The Arthur Smith Institute for Urology at the North Shore-Long Island Jewish Health System. The group will meet every six months to discuss safety issues and review any adverse patient events and if additional issues need to be discussed, meetings will be held on an as needed basis as well per the PI. The board will consist of Dr. Rastinehad (PI) and a three other physicians who are not a part of the protocol. Two will be from the urology department (Dr. Vira and Dr. Weiss or their designees) and the third will be from the department of radiology (Dr. C. Sung or his designee).

**Data Disclosure/Publication/Confidentiality**

Data obtained (clinical information, laboratory tests or other medical data contained in the medical record) will be used only in connection with this clinical study. Confidentiality will be maintained by using patient identification numbers instead of names. The records, including results from the scan and perioperative date, linking a subject’s name with their ID number will be maintained in a locked file cabinet at the Smith institute for Urology at Long Island Jewish Medical Center. Study specific computer information will be stripped of identifying information and password protected. Research data will only be accessible to members of the research team. No individual study results will be shared with subjects or any third party. Data that may be reported in scientific journals will only include general group demographic information.

The de-identified data will be shared with our collaborators as described above. There are three collaborators in this study: Philips Healthcare, National Institutes of Health, and iCAD. The information will include MRI information and correlation with the MR/US fusion biopsy pathology, and general patient information (demographics, Labs (PSA), physical exam, age) No personal identifying information will be shared.

**Costs/Compensation**

Subjects will be responsible for cost associated with their standard of care. Subjects will not receive any payment for their participation.

# Voluntary Participation

Participation in this project is voluntary. Those who do not join the study will not be penalized or lose benefits to which they are entitled. Subjects may withdraw at any time without any consequence. If a subject withdraws from the study after his or her data is already collected, that information may be utilized for research purposes, although the subject will no longer be contacted and no new data will be collected.

References:

1. Needell MH, Slotkin GE, Mitchell FD, Friedman M., Prostatic needle biopsy. J Urol. 1955 Jul;74(1):138-41 [↑](#endnote-ref-1)
2. Pearlman, C. K.: Transrectal biopsy of the prostate. J Urol, 74: 387, 1955 [↑](#endnote-ref-2)
3. Catalona WJ, Smith DS, Ratliff TL, Dodds KM, Coplen DE, Yuan JJ, Petros JA, Andriole GL. Measurement of prostate-specific antigen in serum as a screening test for prostate cancer. N Engl J Med. 1991 Apr 25;324(17):1156-61 [↑](#endnote-ref-3)
4. Cooner, W. H., Mosley, B. R., Rutherford, C. L., Jr. et al.: Prostate cancer detection in a clinical urological practice by ultrasonography, digital rectal examination and prostate specific antigen. J Urol, 143: 1146, 1990 [↑](#endnote-ref-4)
5. M, D. P., Niemann, T. H., Bahnson, R. R.: Extended sector biopsy for detection of carcinoma of the prostate. Urol Oncol, 6: 91, 2001 [↑](#endnote-ref-5)
6. Naughton CK, Miller DC, Mager DE, Ornstein DK, Catalona WJ.: A prospective randomized trial comparing 6 versus 12 prostate biopsy cores: impact on cancer detection. J Urol. 2000 Aug;164(2):388-92. [↑](#endnote-ref-6)
7. Presti JC Jr, O'Dowd GJ, Miller MC, Mattu R, Veltri RW.: Extended peripheral zone biopsy schemes increase cancer detection rates and minimize variance in prostate specific antigen and age related cancer rates: results of a community multi-practice study. J Urol. 2003 Jan;169(1):125-9 [↑](#endnote-ref-7)
8. D'Amico AV, Whittington R, Malkowicz B, Schnall M, Schultz D, Cote K, Tomaszewski JE, Wein A. Endorectal magnetic resonance imaging as a predictor of biochemical outcome after radical prostatectomy in men with clinically localized prostate cancer. J Urol. 2000 Sep;164(3 Pt 1):759-63. [↑](#endnote-ref-8)
9. Fütterer JJ, Heijmink SW, Scheenen TW, Jager GJ, Hulsbergen-Van de Kaa CA, Witjes JA, Barentsz JO.: Prostate cancer: local staging at 3-T endorectal MR imaging--early experience. Radiology, 238: 184, 2006 [↑](#endnote-ref-9)
10. Turkbey B, Albert PS, Kurdziel K, Choyke PL., Imaging localized prostate cancer: current approaches and new developments. AJR Am J Roentgenol. 2009 Jun;192(6):1471-80 [↑](#endnote-ref-10)
11. Anastasiadis AG, Lichy MP, Nagele U, Kuczyk MA, Merseburger AS, Hennenlotter J, Corvin S, Sievert KD, Claussen CD, Stenzl A, Schlemmer HP.: MRI-guided biopsy of the prostate increases diagnostic performance in men with elevated or increasing PSA levels after previous negative TRUS biopsies. Eur Urol, 50: 738, 2006 [↑](#endnote-ref-11)
12. Krieger A, Susil RC, Ménard C, Coleman JA, Fichtinger G, Atalar E, Whitcomb LL. Design of a novel MRI compatible manipulator for image guided prostate interventions. IEEE Trans Biomed Eng. 2005 Feb;52(2):306-13. [↑](#endnote-ref-12)
13. Xu S, Kruecker J, Turkbey B, Glossop N, Singh AK, Choyke P, Pinto P, Wood B: Real-time MRI-TRUS fusion for guidance of targeted prostate biopsies. J.Comput Aided Surg. 2008 Sep;13(5):255-64 [↑](#endnote-ref-13)
14. Xu S, Kruecker J, Guion P, Glossop N, Neeman Z, Choyke P, Singh AK, Wood BJ. Closed-loop control in fused MR-TRUS image-guided prostate biopsy. Med Image Comput Comput Assist Interv Int Conf Med Image Comput Comput Assist Interv. 2007;10(Pt 1):128-3 [↑](#endnote-ref-14)
15. Pinto PA, Chung PH, Rastinehad AR, Baccala AA Jr, Kruecker J, Benjamin CJ, Xu S, Yan P, Kadoury S, Chua C, Locklin JK, Turkbey B, Shih JH, Gates SP, Buckner C, Bratslavsky G, Linehan WM, Glossop ND, Choyke PL, Wood BJ. Magnetic Resonance Imaging/Ultrasound Fusion Guided Prostate Biopsy Improves Cancer Detection Following Transrectal Ultrasound Biopsy and Correlates With Multiparametric Magnetic Resonance Imaging. J Urol. 2011 Aug 15 [↑](#endnote-ref-15)
16. Mohamed Amine Jradi, MD, Mohamed Dridi, MD, Mourad Teyeb, MD, Mokhtar Ould Sidi Mohamed, MD, Ramzi Khiary, MD, Samir Ghozzi, MD, and Nawfel Ben Rais, MD. The 20-core prostate biopsy as an initial strategy: impact on the detection of prostatic cancer, Can Urol Assoc J. 2010 April; 4(2): 100–104. [↑](#endnote-ref-16)
