# Supplementary material for: Comparison of Multiparametric MRI Scoring Systems and the Impact on Cancer Detection in Patients Undergoing MR US Fusion Guided Prostate Biopsies
Source: PLoS One. 2015 Nov 25;10(11):e0143404. doi: 10.1371/journal.pone.0143404 (PMC4659614; doi:10.1371/journal.pone.0143404)
Supplement: S4 File — (DOCX) [file pone.0143404.s004.docx]

Supplemental MRI Report

EXAM: MRI PELVIS WOW CONT

CLINICAL INFORMATION: 64 year old Caucasian male with 2 prior negative prostate biopsies one with HGPIN unknown location. No Family history of prostate cancer and the PSA is 5.6 ng/dl. Last Biopsy was 1/15/2013 (1 year ago).

3T MRI of the prostate is performed using endorectal and 16 channel phased array cardiac coil.

The following sequences were obtained:

Small field-of-view Axial, coronal and sagittal fast spin-echo T2-weighted images using endorectal coil.

Small field-of-view axial T1-weighted images using endorectal coil.

Dynamic Axial T1 prior and following administration of 20 cc of gadolinium.

Large field-of-view axial T1 and T2 weight images of the pelvis.

Axial Diffusion weighted imaging with corresponding ADC maps.

Study was processed using a DynaCad software.

Comparison: none available

FINDINGS:

**Prostate gland:**

Size: 6.0 x 3.8 x 4.4 cm. Volume 52 cc. Location of apex and base on the

Axial T2 images: Series 9 image 9 and image 20 respectively.

Hemorrhage: none.

Transition zone: There is mild central prostatic hypertrophy. There are no suspicious lesions.

Peripheral zone: A suspicious lesion in the right mid gland as described below. The rest of the peripheral zone is unremarkable.

**Lesion #1**

Location: Right mid gland peripheral zone

Zone: 3L

T2 axial series, Slice# range: Series 9, image 14 to 15, Center: Image 14.

Size (Transverse, AP, CC): 7 x 6 x 5 mm.

T2: Dark, PIRAD score 4

Diffusion: Mildly restricted, ADC= 900 x 10-6. Negative B-2000, PIRAD score 3

Enhancement: Early arterial enhancement, Type 3 enhancement curve. Focal, PIRAD + DCE.

Adjacent capsule: Intact.

SQS Overall lesion suspicion score: 4

PI-RAD Suspicion: 4

COMPARISON: none

Capsule: Intact

Neurovascular bundle: Unremarkable

Seminal vesicles: Unremarkable

Lymph nodes: No pelvic adenopathy

Osseous structures: No suspicious lesions identified

Urinary bladder: Unremarkable

IMPRESSION: Suspicious lesion in the right peripheral zone as described above. Lesion is accessible to ultrasound guided fusion biopsy.

SQS Overall Score 4= Clinically significant disease is likely to be present
