# Supplementary figures and images for: Comparison of Multiparametric MRI Scoring Systems and the Impact on Cancer Detection in Patients Undergoing MR US Fusion Guided Prostate Biopsies
Source: PLoS One. 2015 Nov 25;10(11):e0143404. doi: 10.1371/journal.pone.0143404 (PMC4659614; doi:10.1371/journal.pone.0143404)

Supplemental Table 1: MRI Settings


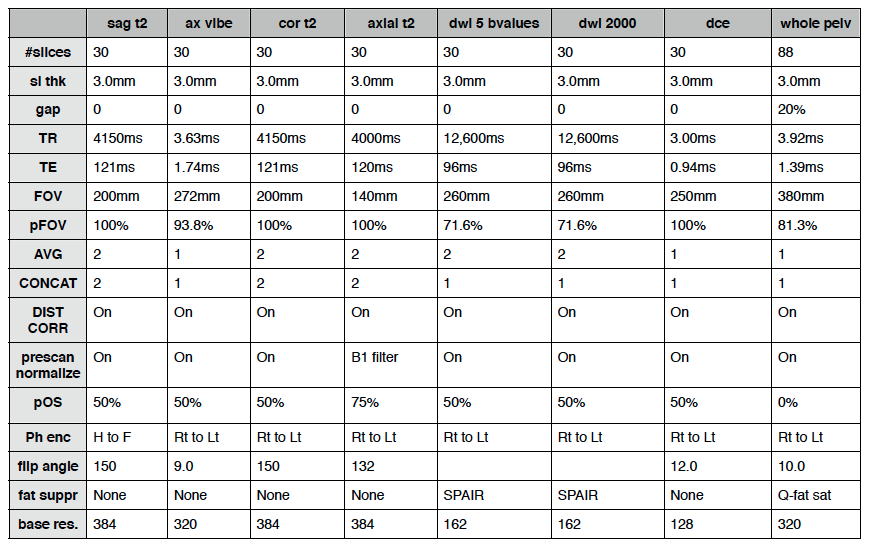


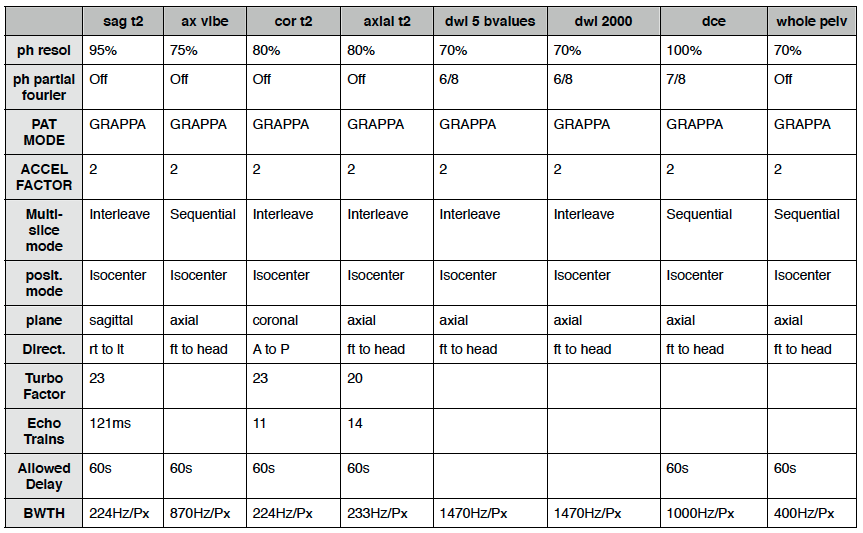

Supplement: S1 Table — (DOCX) [file pone.0143404.s007.docx]
