# Supplementary material for: Comparison of Multiparametric MRI Scoring Systems and the Impact on Cancer Detection in Patients Undergoing MR US Fusion Guided Prostate Biopsies
Source: PLoS One. 2015 Nov 25;10(11):e0143404. doi: 10.1371/journal.pone.0143404 (PMC4659614; doi:10.1371/journal.pone.0143404)
Supplement: S2 File — (PDF) [file pone.0143404.s002.pdf]

North Shore LIJ
North Shore-Long Island Jewish Health System

Institutional Review Board
FWA #00002505
Office of the Institutional Review Board
350 Community Drive, 4th Floor
Manhasset, New York 11030
Phone: 516-719-3100 or 516-562-3100
Fax: 516-562-3105

To: Ardeshir Rastinehad DO
Urology/Radiology
The Arthur Smith Institute
450 Lakeville Road
Lake Success NY 11042

From: Victor Fornari, MD
Chair, Institutional Review Board

Date: Tuesday, February 07, 2012

IRB #: 11-322A
Protocol Title: EMRI/TRUS Fusion Guided Prostate Biopsy - An Improved Way to Detect and Quantify Prostate Cancer . A Phase II Study.
Expiration Date: 11/1/2012

Dear Dr. Rastinehad:

This is to advise you that the submission received 01/05/12 in response to the contingencies set forth by the IRB at their 11/02/2011 meeting, has been reviewed by the Institutional Review Board and the following determination was made:

The above referenced proposal was **APPROVED** by the Institutional Review Board (IRB) at their regularly scheduled meeting on 2/1/2012.

Approval of this project includes the following:

The following investigators are authorized to obtain consent - Ardeshir Rastinehad, Louis Kavoussi, Igor Lobko, and David Siegel.

- Protocol – dated January 4, 2012
- Consent Form – dated January 4, 2012

Subject recruitment methods for enrollment are appropriate, there is equitable selection of subjects, and there are provisions to protect and maintain the confidentiality of data and research participants.

Investigators are reminded that research must be conducted in accordance with all applicable Department of Health and Human Services regulations 45 CFR 46, Food and Drug Administration regulations 21CFR 50, 21CFR 56, 21 CFR 812, and the Health Insurance Portability and Accountability Act (HIPAA). ***All studies are subject to audits by the Office of Research Compliance and/or Institutional Review Board to confirm adherence to institutional, state, and federal regulations governing research. All research studies are expected to conform to Good Clinical Practice (GCP) guidelines.***

NOTE: All IRB Policies and Procedures must be followed, including:

1. Using only IRB-approved consent forms, questionnaires, letters, advertisements, etc. in your research.
2. Submitting any modifications made to the study for IRB review prior to the initiation of changes except when necessary, to eliminate apparent, immediate hazards to the subject.
3. Reporting serious adverse events.

4. Renewing the study at the interval set by the Institutional Review Board (currently 12 months). The expiration date for this study is listed above. You should submit a progress report to the Institutional Review Board at least two months prior to expiration of the study. Failure to receive notification that it is time to renew does not relieve you of your responsibility to provide the IRB with the Progress Report in time for the request to be processed and approved prior to your expiration date.
5. **Prior to implementation, any changes made to studies utilizing the CRC and/or TAP must have CAC and/or COPP, as well as IRB approval.**

IMPORTANT REMINDER: The International Committee of Medical Journal Editors (ICMJE) requires registration of clinical research studies meeting specific guidelines prior to publication. Please see ICMJE requirements for registration of clinical trials at http://www.icmje.org. To register your trial: http://prsinfo.clinicaltrials.gov. You must register your trial PRIOR TO ENROLLING SUBJECTS.
